# On the Mode of Formation of Shells of Animals, of Bone, and of Several Other Structures, by a Process of Molecular Coalescence, Demonstrable in Certain Artificially Formed Products

**Published:** 1860-01

**Authors:** 


### BIBLIOGRAPHICAL NOTICE.

### ARTICLE IX.

*On the Mode of Formation of Shells of Animals, of Bone, and of several other structures, by a process of Molecular Coalescence, demonstrable in certain artificially formed products.* By George Rainey, M. R. C. S., Churchill, London.

Mr. Rainey is already well known to the European profession as one of the most persevering and industrious contributors to the various scientific reviews, and also to the Microscopical Journal, &c. A great variety of subjects are treated of in the work before us, which have of course been described or suggested by others. The author remarks: "As the facts and views to which I allude are for the most part of a general character, and of *dubious* authority, I have thought it better to refrain from mentioning *names* in connexion with them."

If we mistake not, Mr. Rainey successfully combated the strange theory of Mr. Tomes, and others of his school, of the so-called dentinal tubes. The author claims, firstly, a process by which carbonate of lime can be made to assume a globular form, and the explanation of the nature of the process, "Molecular Coalescence," by which the form is produced. Secondly, the explanation of the probable cause of crystallization, and the manner in which the rectilinear form of crystals is effected. Thirdly, the discovery of a process of "molecular disintegration" of the globules of carbonate of lime by inverting the mechanical conditions upon which their previous globular form had depended. Fourthly, the recognition in animal tissues of forms of earthy matter analogous to those produced artificially. And fifthly, the deduction from the above fact, and considerations of the dependence of the rounded forms of organized bodies on physical and not on vital agencies.

"After explaining the process of coalescence, molecular disintegration, the structure of crab-shell, bone, &c., we arrive at that portion of the work which is most interesting and instructive to the dental profession, the dental tubes, &c. The author here remarks upon this subject, "Dr. Carpenter observes, that 'the calcareous layer of the crab-shell is composed of a substance exactly analogous to ivory, being very transparent, and apparently homogeneous, when cut into very thin slices, and being perforated by an immense number of minute sinuous tubuli, which run nearly parallel to one another, from one surface of the shell to the other. This arrangement may be seen by making a thin section of any part of the shell; but it may be shown particularly well in the end of the claw, which is thicker and of denser texture than the rest. A transverse section of this shows the tubes radiating from the central cavity towards the external surface, and would, I feel assured, be regarded by the most experienced observer as the section of a tooth if he were not informed of its real nature.' The resemblance between dentine and crab-shell, as observed by Dr. Carpenter, is in most respects correct and sufficiently striking to justify the inference that they are formed in a similar manner. This, I believe, is the general opinion of physiologists. The globular particles of carbonate of lime in shell are doubtless analogous to the globular dentine in teeth, and the apparent spaces between the radiating lines in the former correspond in a great measure to the so-called dentinal tubes in the latter. But respecting the fact of these being, in either case, tubes, the results of my experience and investigations are opposed to this view. It is evident to me from the plate xv, fig. 14, in the descriptive catalogue of the Royal College of Surgeons, that if the tubes there represented are intended to show a form of structure analogous to dentinal canals as they are generally termed, the author has made a great mistake, I may observe, that in the end of the crab's claw, as seen by the microscope, there are, as in other parts, the two different kinds of structure just described, one consisting of alternate dark and light generally sinuous lines, extending from the superficial towards the deep surface of the shell generally considered to be tubular. These lines exist in every part of the shell sufficiently thick to present them and in certain positions, as in the end of the claws, their course gives them very much the appearance of dentine. However, I may repeat the observation, that, in the claw, especially of the very young animal, they degenerate into mere dots before they arrive at the cavity of the claw and present nothing in their appearance of a tubular character. The cause of this arrangement of the particles of the shell has been explained, and there can be little doubt but that this same explanation applies to the spaces existing between the longitudinal portions of dentine. These latter spaces are in principle analogous to the canaliculi of bone, which will be fully explained in the article on bone. Hence, in one form of bone the cementum or crusta petrosa, canaliculi and dentinal canals sometimes exist together. The dentinal canals are merely spaces of feeble or imperfect cohesion continued from the interglobular spaces where such exist between longitudinal portions of coalesced dentine to the pulp cavity. These passages becoming a little widened from the contraction of the part in drying and containing air, seem to have, under the microscope, especially if appearances depending upon distance are not sufficiently allowed for the appearance of tubes. It is, however incompatible with the function of tubes, as they exist in other parts, that a system of such organs intended for the conveyance of secreted fluids should be simple prolongations of the interstices of the body as are the spaces between globular portions of dentine and the pulp-cavity of a tooth, this latter, notwithstanding its size, being only a cellular interval. The other structure is distinctly tubular. It does not exist excepting in the tegumentary part of the shell, and it is, which is most unquestionably represented in the plate before referred to. These tubes pass from the external surface of the shell, through its substance to its deep one."

The preceding extract from the work clearly shows the opinion of the author as bearing on Mr. Tomes' theory upon the subject of "dentinal tubes." The dental profession will feel much indebted to the author of this little work for clearing away the cobwebs of a false theory—and we trust ere long this valuable contribution to science will find a place in every dental library on this side of the Atlantic.

